# Leadership skills training in Psychiatry: A European-based cross-sectional survey

**DOI:** 10.1192/j.eurpsy.2022.265

**Published:** 2022-09-01

**Authors:** M. Santos, A. Samouco, Z. Azvee, D. Krishnan, N. Zaja, J. Pedro, R. Maitra, A. László, M. Fellinger, U. Halbreich, M. Pinto Da Costa

**Affiliations:** 1 Hospital Prof. Doutor Fernando Fonseca, Department Of Psychiatry, Amadora, Portugal; 2 Unidade Local de Saúde do Norte Alentejano, Department Of Psychiatry And Mental Health, Portalegre, Portugal; 3 Mater Misericordiae University Hospital, Department Of Psychiatry, Dublin, Ireland; 4 University of Nottingham, Nottinghamshire Healthcare NHS Foundation Trust, Institute Of Mental Health, Nottingham, United Kingdom; 5 University Psychiatric Hospital Vrapče, Department For Biological Psychiatry And Psychogeriatrics, Zagreb, Croatia; 6 Tavistock and Portman NHS Foundation Trust, Child And Adolescent Psychiatry, London, United Kingdom; 7 Arad County Emergency Clinical Hospital, Department Of Psychiatry, Arad, Romania; 8 Medical University of Vienna, Department Of Psychiatry And Psychotherapy, Vienna, Austria; 9 University at Buffalo - The State University of New York at Buffalo (SUNY-AB), Jacobs School Of Medicine And Biomedical Sciences, Buffalo, United States of America; 10 King’s College London, Institute Of Psychiatry, Psychology & Neuroscience, London, United Kingdom

**Keywords:** training, leadership, psychiatry, skills

## Abstract

**Introduction:**

Leadership in healthcare organisations is crucial to continually improve and provide high quality compassionate care. Leadership development and training enables the psychiatrists in developing these essential skills. Focusing on how to enhance leadership development through leadership skills training and experiential learning should be a priority. However, little is known about the extent to which this leadership skills training is available across Europe in the early stage of the career of psychiatrists.

**Objectives:**

To investigate the access to leadership development opportunities among European psychiatric trainees and early career psychiatrists (ECPs) and their perceptions related to leadership skills training.

**Methods:**

Cross-sectional study, using an online survey consisting of multiple-choice questions and free text responses.

**Results:**

Participants from 33 European countries took part in this survey, where the majority were female. More than half were general adult psychiatric trainees and more than a quarter ECPs. About half indicated having no access to leadership skills training within their training program, with only about 10% being satisfied with the training received. About half sought additional training outside their program. A vast majority requested training in leadership skills to be included in a psychiatric training program.

**Conclusions:**

Our study provides an overview of important gaps in availability and access to leadership skills training amongst psychiatric trainees and ECPs across Europe. We hope that this study will help inform future actions pertaining to development and improvement of leadership skills training for trainees and ECPs across Europe.

**Disclosure:**

No significant relationships.

